# A rat study on the PTEN expression in ovarian tissue in PCOS and folliculogenesis

**DOI:** 10.1038/s41598-023-47809-y

**Published:** 2023-11-26

**Authors:** Muberra Namlı Kalem, Elvan Anadol, Ziya Kalem, Perihan Yalçınkaya Sezginer, Cigdem Elmas, Canan Yılmaz, Batuhan Bakirarar

**Affiliations:** 1 Obstetrics and Gynecology Department, Istinye University, Istanbul, Turkey; 2 Laboratory Animal Breeding and Experimental Researches Center, Gazi University, Ankara, Turkey; 3 Department of Histology and Embryology, Alaaddin Keykubat University, Antalya, Turkey; 4 Department of Histology and Embryology, Gazi University, Antalya, Turkey; 5 Department of Biochemistry, Gazi University, Ankara, Turkey; 6 Department of Biostatistics, Ankara University, Ankara, Turkey

**Keywords:** Endocrine reproductive disorders, Infertility, Experimental models of disease

## Abstract

The objective of this investigation was to examine alterations in PTEN expression within ovarian tissue in a rat model of polycystic ovary syndrome (PCOS). The analysis also encompassed the examination of PTEN alterations in the ovarian tissue throughout the process of folliculogenesis in rats with normal ovulatory cycles. The study involved 12 adult female Sprague‒Dawley rats randomly assigned to the letrozole-induced polycystic ovary syndrome (PCOS) group as part of an animal-based research endeavour. The sections derived from the ovaries were subjected to immunohistochemical staining for PTEN. The evaluation of PTEN staining levels in ovarian tissues was conducted using electron microscopy. Follicle counts, as well as hormonal and biochemical analyses (serum luteinising hormone (LH), follicle-stimulating hormone (FSH), anti-Müllerian hormone (AMH), testosterone, oestradiol levels and serum glucose, triglyceride, HDL and LDL-cholesterol levels), were conducted to provide evidence of the manifestation of polycystic ovary syndrome (PCOS) in rats. The number of primordial and Graafian follicles in the PCOS group decreased significantly, and the number of primary, secondary and antral follicles increased significantly. PTEN expression was found to be significantly higher in the PCOS group than in the control group in the primordial follicle oocyte cytoplasm, primordial follicle granulosa cells, primary follicle oocyte cytoplasm, primary follicle granulosa cells, antral follicle oocyte cytoplasm, antral follicle granulosa cells, and corpus luteum (p** = **0.007, p** = **0.001, p** = **0.001, p** = **0.001, p** = **0.001, p** = **0.002, and p** = **0.018, respectively). In the non-PCOS group, a time-dependent comparison of the amount of oocyte cytoplasm and PTEN staining in granulosa cells of the oocytes at different stages of development was performed. While the follicles were developing from the primordial follicle to the primary and antral follicle, the amount of PTEN staining in the oocyte cytoplasm decreased, whereas the PTEN activity in the granulosa cells increased as the oocyte developed (p = 0.001 and p = 0.001, respectively). The current investigation demonstrated changes in PTEN expression in ovarian tissue throughout the course of normal folliculogenesis, as well as in instances of disrupted folliculogenesis, with a focus on rats with PCOS.

## Introduction

PCOS, a prevalent endocrine condition affecting women of reproductive age^1^, is a complex disorder with various clinical manifestations and associated morbidities^2^. The prevalence varies across different regions, with rates ranging from 6 to 21%, according to the ESHRE/ASRM 2003 criteria^3^. Despite extensive research, the precise molecular pathways involved in PCOS pathogenesis remain unexplored, highlighting the need for further analyses and treatments^4^.

PCOS affects the development of a mature oocyte, which is a crucial part of human follicle development^5^. Primordial follicles, grouped together as a cohort, are recruited for ovulation of a mature oocyte^6^. The intrafollicular hormonal environment is dynamic, and effective communication between granulosa cells and oocytes determines the oocyte’s developmental competence. Granulosa cells surrounding the oocyte play a crucial role in supplying nutrients and growth factors for oocyte development^7^. In PCOS, although there are more follicles in the ovary than in that without PCOS, a mature oocyte is challenging to form due to impaired follicle developmental stages^8^. Granulosa cells that surround oocytes in PCOS exhibit abnormal patterns of proliferation and apoptosis, which can be attributed to processes such as atresia, degeneration, and hypertrophy^9^.

PTEN (phosphatase and tensin homologue) is a tumour suppressor gene that plays a crucial role in regulating cell growth and division^10^. PTEN regulates the Akt/PKB signalling pathway, affecting phosphoinositol triphosphate levels, which are essential for cell development and cell survival^11^. The PI3K/AKT signalling pathway can affect ovarian granulosa cells, and the PI3K catalytic subunit p110d has been shown to be an important component of the PI3K pathway for follicular development stimulated by both follicle-stimulating hormone and oestradiol in ovarian granulosa cells^12,13^. PTEN deletion on the tenth chromosome dephosphorylates PIP3 to PI and P2, inhibiting the PI3K/AKT pathway and slowing biological processes, including proliferation, growth and motility^14^.

The impairment of PTEN function can result in detrimental consequences for the intricate hormonal signalling equilibrium within the ovaries, specifically with regards to folliculogenesis in PCOS. This phenomenon can be attributed to the involvement of PTEN in the regulation of ovarian follicle growth and oocyte maturation^15^. The PTEN protein, acting as a regulatory factor, exerts a substantial influence on both the proliferation and differentiation of granulosa cells and plays a critical role in the maturation of oocytes, facilitating the coordination of essential events needed for successful and optimal ovulation^16^.

In this study, PTEN expression in ovarian tissue was compared in a PCOS rat model and normo-ovulatory rats. PTEN tissue changes in a time-dependent manner during folliculogenesis in normo-ovulatory rats were also shown.

## Materials and methods

### Animals and experimental design

For the purpose of our research, a total of 12 female adult Sprague‒Dawley rats weighing 160–180 g were acquired from Harlan Laboratories located in the Netherlands. These rats were subjected to a 12-h light and dark cycle and had unrestricted access to a standard rat diet sourced from Korkuteli Food Industry in Turkey. The female rats, which exhibited regular oestrous cycles as confirmed by vaginal cytologic examination, were randomly allocated into two distinct groups. Aromatase inhibitor (Femara tablet; letrozole 2.5 mg, Novartis) dissolved in 1% carboxymethyl cellulose (CMC) (2 ml/kg) was administered daily through oral gavage at 1 mg/kg/day to Group 1 consisting of 6 rats for 21 days (the PCOS group), and 1% CMC (2 ml/kg) solution was administered daily through oral gavage to Group 2 (the control group) for 21 days. The methodology and dosage of the drug were established based on prior studies documented in the literature, wherein PCOS was induced in rats using letrozole^17–19^.

The sexual cycle was determined by microscopic analysis of the predominant cell type in vaginal smears obtained daily until the end of the experiment. All control rats had a normal oestrous cycle of 4.5 days, whereas letrozole-treated rats were completely acyclic, and vaginal smears showed leukocytes, the predominant cell type of the diestrus phase. Hormonal and histological changes in the ovary and uterus in both the control group and the letrozole group were evaluated in line with the data obtained as a result of vaginal cytological examination. All rats in the control group were sacrificed in the proestrus phase of the oestrous cycle.

All procedures that used animals were approved by the Gazi University Animal Experiments Local Ethics Committee in Turkey and were performed at the Laboratory Animal Breeding and Experimental Research Center of the same university. All animals were sacrificed with ketamine (45 mg/kg, i.m.) and xylazine (5 mg/kg, i.m.) anaesthesia. Female reproductive tissues (uterus and ovaries) were excised, and the dry weights of the ovaries were determined with a precision digital scale sensitive to 0.01 g. Tissues were first fixed for 72 h in a 10% neutral formaldehyde solution for light microscopic examination, and paraffin blocks were provided using routine procedures.

Blood samples were drawn intracardially into plain tubes without heparin on the 22nd day of administration for endocrine and biochemical analyses, and serum was separated by centrifugation at 3000 rpm for 10 min and stored at − 80 °C until analysis.

### Hormonal and biochemical analysis

Serum luteinising hormone (LH), follicle-stimulating hormone (FSH), anti-Mullerian hormone (AMH), testosterone and oestradiol levels were estimated by enzyme-linked immunosorbent assays. FSH and LH analyses were performed using an ELISA kit (SunRed; SunRed Biotechnology Company Shanghai China), AMH analyses were performed with an ELISA kit (Ansh Labs, AL-113), and testosterone and oestradiol levels were determined by an ELISA kit (Oxford; Serotec, Ltd., UK). Serum glucose, triglyceride, HDL and LDL-cholesterol levels were measured by enzymatic methods using autoanalysers.

### Histology

The right ovary of all rats was bisected longitudinally**.** Fixation of the ovarian tissues was carried out in 10% formalin solution, followed by routine tissue processing. Sections were obtained with a thickness of 5 μm and placed on glass slides. Sampling of consecutive sections was chosen systematically and randomly. These sections were stained with haematoxylin and eosin and then photographed using a Leica DCM 4000 microscope and an HD digital camera. Finally, stereological and histopathological investigations were carried out on micrographs.

### Haematoxylin and eosin staining

Sections obtained from the ovaries were maintained in an incubator at 37 °C overnight. The paraffin sections were deparaffinized in xylene and rehydrated. Then, the specimens underwent a dehydration process with a graded series of ethanol (EtOH). The sections were then incubated in haematoxylin solution for 10 min and washed again under running water for 10 min. Next, the sections were dipped 2–3 times in a mixture of 70% alcohol plus three drops of glacial acetic acid, washed in running water for 10 min and stained with eosin solution for 5 min. Finally, the sections were washed in running water for 10 min, passed through 3 changes of 95% EtOH and 2 changes of 100% EtOH for 1 min each and cleared in 3 changes of xylene for 1 min each^20^. Coverslipped images were taken and evaluated on a Leica DCM 4000 computer-aided imaging system.

### Stereology (follicle counting)

Physical disector and Cavalieri methods were used to estimate the number of ovarian follicles and mean volume of regions of interest in ovarian tissues^21^. The mean ovarian tissue volume was determined by multiplying the area of a point interval by the sum of the number of points that intersect the region of interest in each section. The mean volumes of the regions of interest were calculated using the following formula: Volume (V1) = t × A, where “t” represents the thickness and interval of each section, and “A” is the total area of the regions of interest. The sampling and counting methodology was devised based on a preliminary pilot study, and particles were enumerated using the ImageJ software program. The numerical density of follicles was determined using the following formula: Nv = ∑**Q** − ∑**V** **disector**. The morphological classification of follicles in the ovary was confirmed through the validation of the coefficient of error and coefficient of variation.

The morphological classification of follicles in the ovary was as follows: primordial follicle, unilaminar primary follicle, multilaminar primary follicle, antral follicle, Graafian follicle and corpus luteum^22^.

### Immunohistochemistry (IHC) staining for PTEN

Sections obtained from the ovaries were maintained in an incubator at 37 °C overnight. The study involved deparaffinization of ovarian tissues using various methods. First, the sections were left in xylol for 5 min and then dehydrated in a graded alcohol series for 3 min each. The sections were then fixed in EDTA buffer in a microwave for 10 min to remove formaldehyde. After cooling to 25 °C, tissues were circled with a Pap pen, washed with PBS three times, treated with 3% H2O2, and treated with Ultra-V block. The tissues were then subjected to PTEN primary antibody at 4 °C overnight, followed by treatment with a biotinylated secondary antibody and streptavidin peroxidase for 20 min. Nuclear staining was achieved using 4′,6-diamino-2-phenolindole (DAPI), and background staining was performed with haematoxylin. The slides were closed using Entellan, and image analysis was performed using the Leica DCM 4000 computer-aided imaging system.

### Evaluation of PTEN expression in ovarian sections

PTEN expression was scored using an immunoreactive scoring scale and evaluated by two researchers who did not have any prior knowledge of the groups of rats. Accordingly, six zones (one central and five peripheral) were selected from ovarian tissue sections (5 μm thickness) and subjected to IHC staining with anti-PTEN antibodies. The HSCORE, defined below, was used to evaluate the immunoreactive density in these zones. The HSCORE was determined by the following formula: HSCORE ¼ Pi (i + 1), where ‘i’ was the intensity of labelling with a value of 0, 1, 2 or 3 (none, weak, moderate or strong) and Pi the percentage of labelled cells for each intensity, within a range of 0–100%^18^. The rate of positive cells was scored by the extent of immunostaining and was assigned to one of the following categories: 0 (0%, no positive cells), 1 (≤ 30% positive cells), 2 (30–60% positive cells) and 3 (> 60% positive cells)^18^.

### Statistical analysis

For the data analysis, the SPSS 11.5 program was used. Mean ± standard deviation and median (minimum–maximum) were used as descriptors for quantitative variables, and number (percentage) was used for qualitative variables. If normal distribution assumptions were provided, Student’s t test was used to determine whether there was a difference between the qualitative variable categories and the two quantitative variable categories; the Mann‒Whitney U test was used if they were not. Repeated measures ANOVA was used to determine whether there was a statistically significant difference between quantitative repeated measurements. The Tukey test was used as the post hoc test. The statistical significance level was taken as 0.05.

### Ethical approval

This study was carried out in compliance with the ARRIVE guidelines and all methods were carried out in accordance with relevant guidelines and regulations.

## Results

Rats administered letrozole for the induction of PCOS showed significant increases in ovarian size, and the mean ovarian weight in the PCOS group was found to be approximately twice that of the control group (p < 0.001) (Table 1).

**Table 1:** Comparisons between the control and PCOS groups for follicle counts and ovarian weights.

Variables	Control group (n = 6)		PCOS group (n = 6)		p-value
	Mean ± SD	Median (Min–Max)	Mean ± SD	Median (Min–Max)	
Ovarian weights (gr)	0.08 ± 0.01	0.08 (0.07–0.10)	0.16 ± 0.02	0.16 (0.13–0.18)	< 0.001
Primordial follicle (n)	48.00 ± 7.62	47.38 (37.50–59.00)	16.88 ± 3.45	17.13 (11.25–22.00)	< 0.001
Unilaminar primary follicle (n)	6.54 ± 0.53	6.50 (5.75–7.25)	12.75 ± 0.69	12.63 (12.00–13.75)	< 0.001
Multilaminar primary follicle(n)	5.00 ± 1.08	4.75 (3.75–6.75)	9.79 ± 0.81	9.63 (9.00–11.00)	< 0.001
Antral follicle (n)	2.29 ± 0.19	2.25 (2.00–2.50)	7.29 ± 0.87	7.25 (6.25–8.50)	< 0.001
Graafian follicle (n)	2.67 ± 0.34	2.75 (2.25–3.00)	1.83 ± 0.30	1.88 (1.50–2.25)	0.001

SD, standard deviation; Min, minimum; Max, maximum; student t-test was used.

In the process of follicle counting, a sample size of 48 sections was utilised, with each rat contributing one ovary and four sections being taken from each ovary.

### Histopathological findings

The follicle numbers of the control and PCOS groups in different developmental stages were compared after H&E staining. Table 1 summarises the comparisons between the control and PCOS groups for follicle numbers. The PCOS group had significantly lower primordial follicle and Graaf follicle counts than the control group (p** < **0.001). Furthermore, the PCOS group had significantly higher unilaminar primary follicle, multilaminar primary follicle, and antral follicle numbers than the control group (p < 0.001) (Table 1).

### Hormones and biochemical findings

Comparisons between the control and PCOS groups for hormones and biochemical findings are summarised in Table 2. LH, FSH, and oestrogen levels in the PCOS group were found to be significantly lower than those in the control group (p** = **0.012, p** < **0.001, and p** = **0.001, respectively). Furthermore, AMH, testosterone, triglyceride, glucose, and LDL levels in the PCOS group were found to be significantly higher than those in the control group (p** = **0.003, p < 0.001, p** = **0.015, p = 0.001, and p = 0.002, respectively).

**Table 2:** Comparisons between control and PCOS groups for hormones, glucose and lipids.

Variables	Control group (n = 6)		PCOS group (n = 6)		p-value
	Mean ± SD	Median (Min–Max)	Mean ± SD	Median (Min–Max)	
LH (IU/L)	0.22 ± 0.02	0.22 (0.20–0.26)	0.19 ± 0.02	0.19 (0.16–0.21)	**0.012**
FSH (mIU/ml)	13.17 ± 2.38	12.74 (10.51–17.57)	5.48 ± 1.44	5.10 (3.68–7.90)	** < 0.001**
AMH (ng/ml)	2.42 ± 0.38	2.43 (1.90–2.94)	3.55 ± 0.55	3.33 (3.03–4.31)	**0.003**
Estrogen (pg/ml)	80.58 ± 7.01	81.33 (69.73–88.47)	48.32 ± 13.05	48.76 (31.36–62.59)	**0.001**
Testosterone (ng/dl)	7.33 ± 0.57	7.24 (6.70–8.30)	14.47 ± 2.06	15.13 (11.77–16.50)	** < 0.001**
Total cholesterol (mg/dl)	42.83 ± 8.38	43.00 (32.00–53.00)	43.17 ± 6.85	42.50 (36.00–53.00)	0.941
HDL (mg/dl)	29.75 ± 5.58	29.45 (20.80–35.80)	24.32 ± 4.24	23.10 (19.80–31.10)	0.087
Triglyceride (mg/dl)	41.38 ± 15.20	40.20 (26.00–62.10)	132.48 ± 62.32	118.40 (52.20–230.40)	**0.015**
Glucose (mg/dl)	179.83 ± 10.33	180.60 (163.40–191.70)	249.33 ± 37.41	242.40 (203.90–317.20)	**0.001**
LDL (mg/dl)	6.67 ± 0.82	6.50 (6.00–8.00)	11.17 ± 2.14	11.00 (9.00–14.00)	**0.002**

SD, standard deviation; Min, minimum; Max, maximum; student t-test was used.

Significant values are in [bold].

### Immunohistochemical findings for the ovary

**Figure 1:**
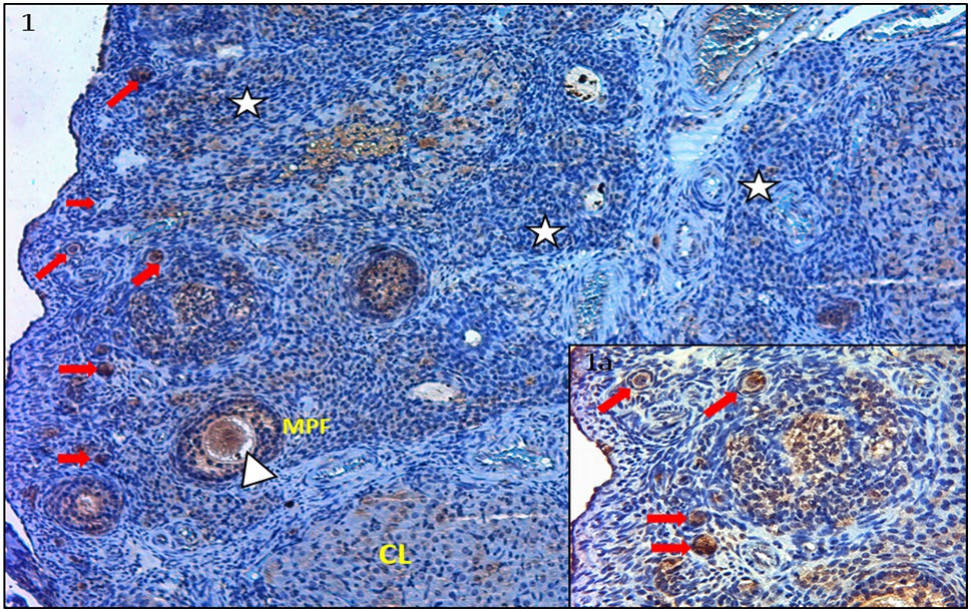
IHC staining with PTEN primary antibody of ovarian tissues in the control group; Primordial follicles (red arrows), the multilaminar primary follicle (MPF), oocyte cytoplasm (white arrows), corpus luteum (CL), stroma (white star) (× 100). (**Ia)** Primordial follicles (red arrows) (× 400). Strong PTEN involvement was observed in the oocyte cytoplasm of the primordial follicle, and weak involvement in the granulosa cell cytoplasm surrounding the follicle. A moderate uptake was observed in the granulosa cell cytoplasm of the primary follicle growing in the surrounding area. Corpus Luteum involvement was moderate.

**Figure 2:**
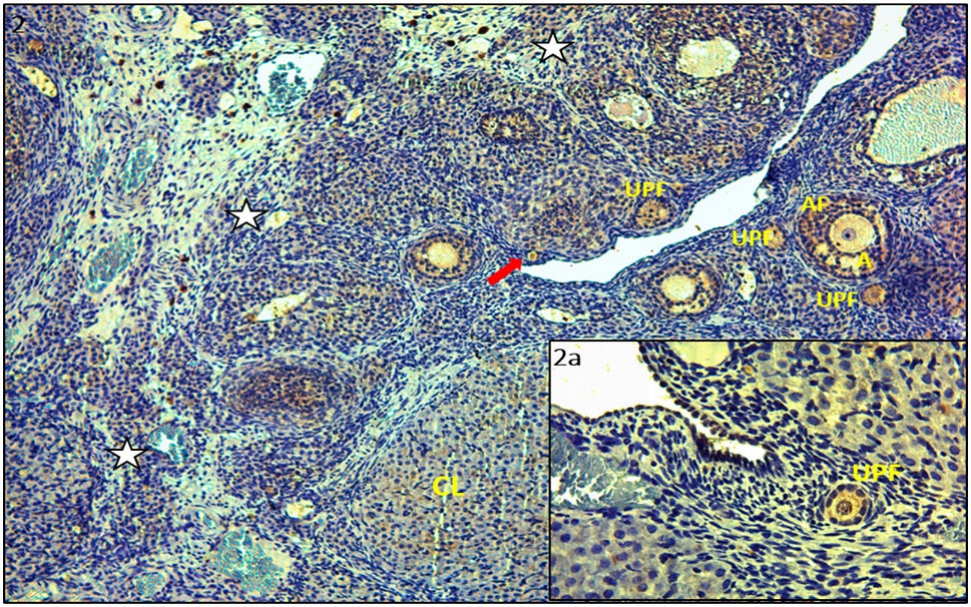
IHC staining with PTEN primary antibody of ovarian tissues in the control group; Primordial follicles (red arrows), the unilaminar primary follicle (UPF), the antral follicle (AF), corpus luteum (CL), stroma (white star) (× 100). (**IIa**) Unilaminar primary follicle (UPF) (× 400). PTEN involvement was strong in the oocyte cytoplasm of the primordial follicle, weak in the granulosa cell cytoplasm of the primordial follicle, and strong in the granulosa cells of the secondary and antral follicles. PTEN immune reactivity was moderate in the granulosa cells of the unilaminar primary follicle.

**Figure 3:**
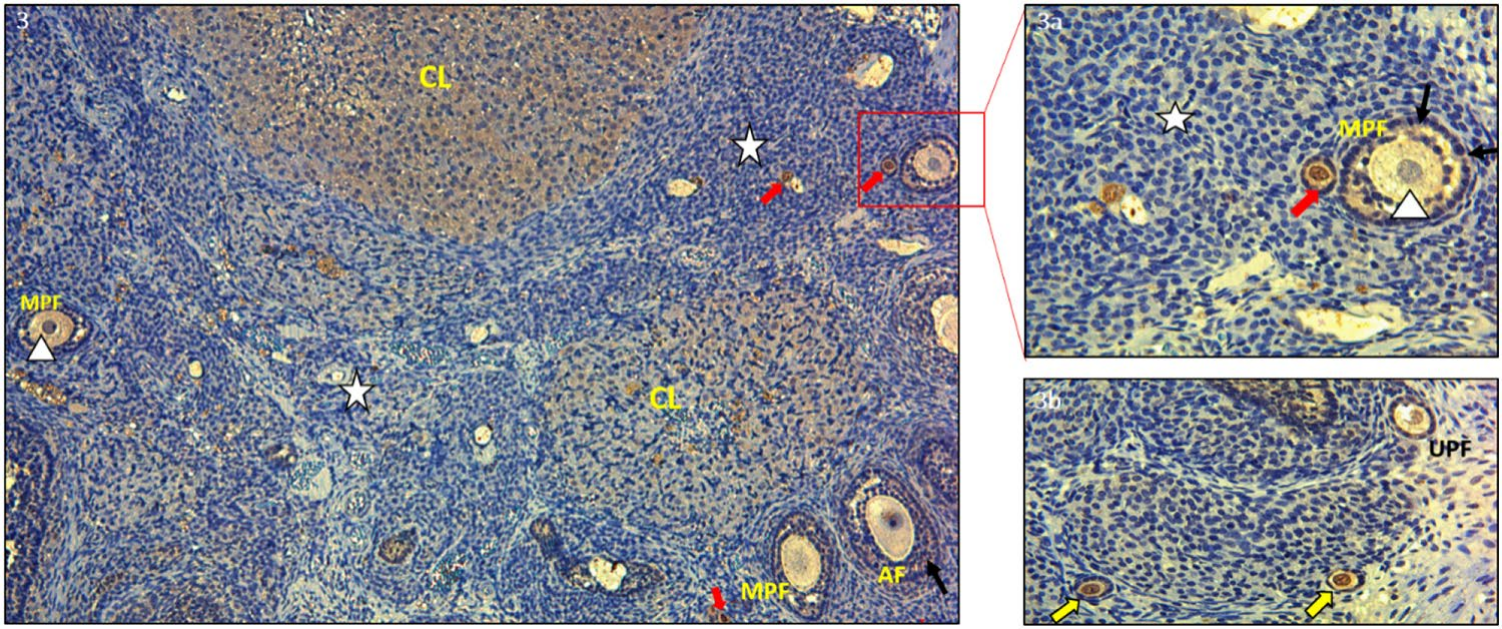
In the ovarian tissue of the control group stained with PTEN primary antibody; primordial Follicle (), Multilaminar Primary Follicle (MPF), Antral Follicle (AF), Oocyte Cytoplasm (), Corpus Luteum (CL), Stroma () (DAP—Hematoxylin × 100). (**IIIa**) Primordialfolicula (), Multilaminar Primer Follicle (MPF), Oocyte Cytoplasm () (DAP—Hematoxylin × 400). (**IIIb**) Primordial Follicle- Primary Follicle Transition () Unilaminar Primary Follicle (UPF) (DAP—Hematoxylin × 400). PTEN immunoreactivity was strong in the oocyte cytoplasm of the **primordial follicle**, weak in the granulosa cells surrounding the oocyte, and varying from weak to moderate in the oocyte cytoplasm of the cubic granulosa cells. PTEN involvement was moderate in the surrounding unilaminar primary follicle granulosa cells and moderate to strong in the multilaminar primary and antral follicle granulosa cells. In the corpus luteum, PTEN immunoreactivity was moderate.

**Figure 4:**
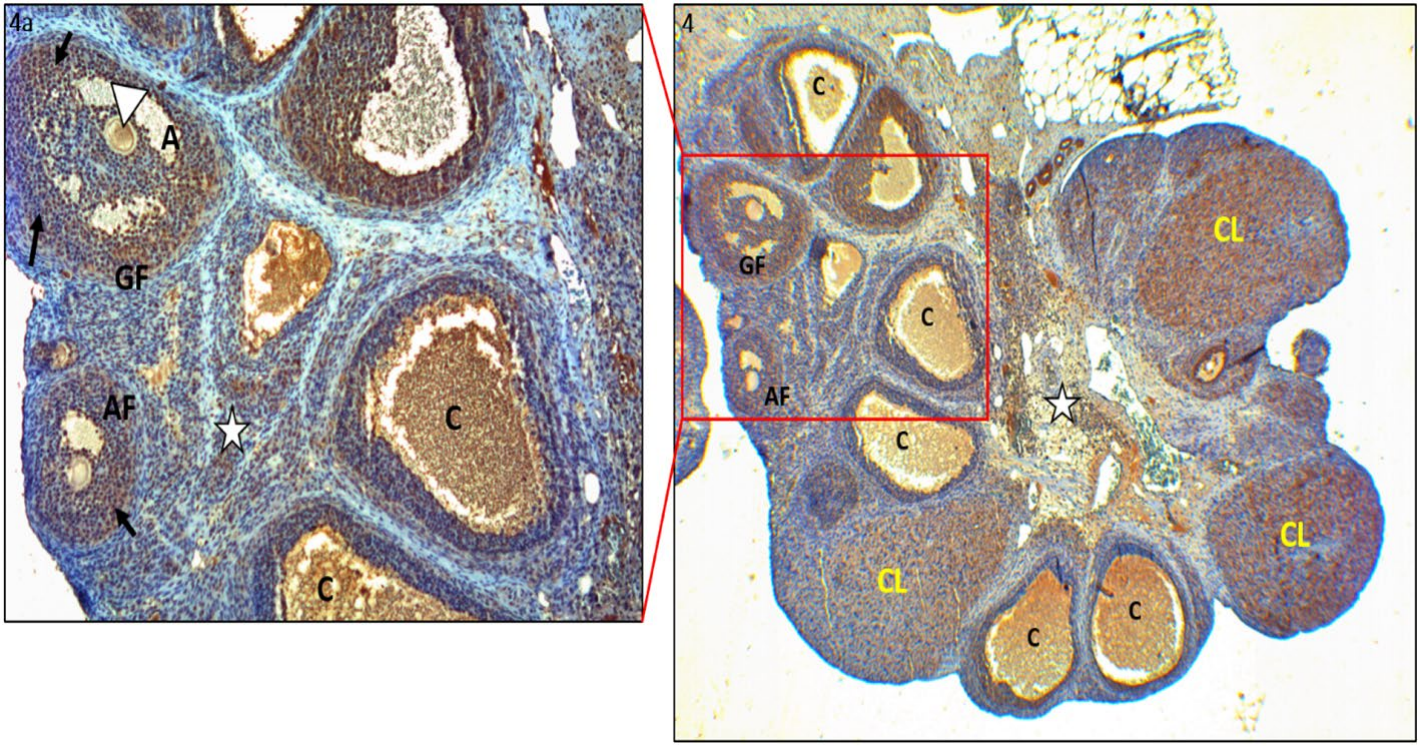
IHC staining with PTEN primary antibody of ovarian tissues in the PCOS group; Antral follicle (AF), Graaf follicle (GF), corpus luteum (CL), Cyst (C), stroma (white star) (× 100). (**Iva**) Antral follicle (AF), Graaf follicle (GF), oocyte cytoplasm (white arrows), granulosa cells (black arrows), Cyst(C), stroma (white star) (× 400). Moderate PTEN involvement was observed in the oocyte cytoplasm of the antral and Graafian follicles and strong PTEN involvement was observed in the granulosa cells surrounding the oocytes in the PCOS group. Once more, strong PTEN immunoreactivity was observed in both cystic structures and the Corpus Luteum.

**Figure 5:**
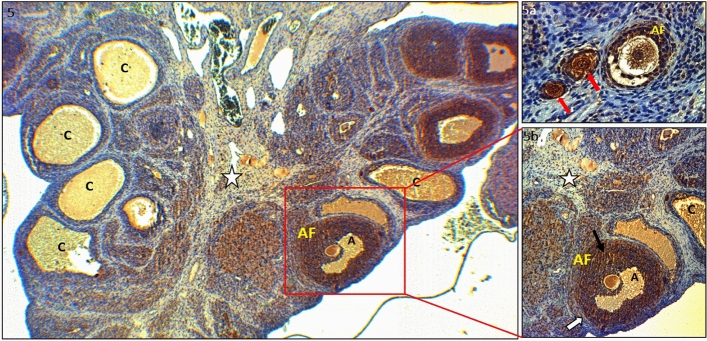
IHC staining with PTEN primary antibody of ovarian tissues in the PCOS group; Antral follicle (AF), antrum (A), cyst (C), stroma (white star) (× 100). (**Va**) Antral follicle (AF), the unilaminar primary follicle (UPF) (× 400). (**Vb**) Antral follicle (AF), granulosa cells (black arrows), Cyst(C), stroma (star) (× 400). Moderate PTEN involvement was observed in the oocyte cytoplasm of the antral follicle in the PCOS group, whereas strong PTEN involvement was observed in the granulosa cells surrounding the oocyte. Additionally, strong PTEN immunoreactivity was observed in both cystic structures and the Corpus Luteum. PTEN involvement in primordial and primary follicles was quite strong.

As a result of IHC staining, PTEN primary antibody immunoreactivity was detected at every stage of follicular development. PTEN expression was observed in oocyte cytoplasm, granulosa cells, theca cells and the corpus luteum of follicles belonging to the control group (Figs. 1, 2, 3) and PCOS group (Figs. 4, 5).

PTEN expression was strong in the oocyte cytoplasm of primordial follicles but weak in granulosa cells (Fig. 1 and a). Similarly, PTEN immunoreactivity was moderate in the granulosa cells surrounding the oocyte in unilaminar primary follicles, but it was significantly strong in the granulosa cells belonging to multilaminary primary follicles and antral follicles (Fig. 2 and a). In the control group, during the transition from the primordial follicle to the primary follicle, PTEN immunoreactivity was observed to be strong in the primordial follicle cytoplasm, weak in the squamous granulosa cell surrounding the oocyte, and varying from weak to moderate in the oocyte cytoplasm with cubic granulosa cells (Fig. 3, a and b).

In the PCOS group, moderate PTEN immunoreactivity was observed in the oocyte cytoplasm of the antral and Graafian follicles, but this expression was strong in the granulosa cells of these oocytes. PTEN immunoreactivity was also strong in the cystic structures and in the corpus luteum (Fig. 4 and a). The PTEN immunoreactivity of the primordial and primary follicles was quite strong compared to that in the control (Fig. 5, a and b).

PTEN expression in the control and PCOS groups is presented in Table 3. PTEN staining levels were found to be significantly higher in the PCOS group than in the control group in the primordial follicle oocyte cytoplasm, primordial follicle granulosa cells, primary follicle oocyte cytoplasm, primary follicle granulosa cells, antral follicle oocyte cytoplasm, antral follicle granulosa cells, and corpus luteum (p** = **0.007, p** = **0.001, p** = **0.001, p** = **0.001, p** = **0.001, p** = **0.002, and p** = **0.018, respectively).

**Table 3:** Comparisons between the control and PCOS groups for PTEN staining in ovarian tissues in different stages of folliculogenesis.

PTEN staining levels	Control group (n = 6)		PCOS group (n = 6)		p-value
	Mean ± SD	Median (Min–Max)	Mean ± SD	Median (Min–Max)	
Primordial follicle cytoplasm of oocyte	1.33 ± 0.52	1.00 (1.00–2.00)	2.67 ± 0.52	3.00 (2.00–3.00)	0.007
Primordial follicle granulosa of oocyte	1.00 ± 0.00	1.00 (1.00–1.00)	3.00 ± 0.00	3.00 (3.00–3.00)	0.001
Primary follicle cytoplasm of oocyte	1.00 ± 0.00	1.00 (1.00–1.00)	3.00 ± 0.00	3.00 (3.00–3.00)	0.001
Primary follicle granulosa of oocyte	1.00 ± 0.00	1.00 (1.00–1.00)	3.00 ± 0.00	3.00 (3.00–3.00)	0.001
Antral follicle cytoplasm of oocyte	1.00 ± 0.00	1.00 (1.00–1.00)	3.00 ± 0.00	3.00 (3.00–3.00)	0.001
Antral follicle granulosa of oocyte	1.33 ± 0.52	1.00 (1.00–2.00)	3.00 ± 0.00	3.00 (3.00–3.00)	0.002
Corpus luteum	1.83 ± 0.41	2.00 (1.00–2.00)	2.67 ± 0.52	3.00 (2.00–3.00)	0.018

SD, standard deviation; Min, minimum; Max: maximum; Mann–Whitney U test was used.

PTEN staining levels in both groups of rats were evaluated using an immunoreactive scoring scale, a total of six zones (consisting of one core zone and five outer zones) were chosen from the sections of ovarian tissue.

In the final section of the study, PTEN staining levels in the primordial follicle, primary follicle, and antral follicle stages were compared to demonstrate the change in PTEN immunoreactivity in the follicular developmental stages in the control group. These findings are shown in Table 4. A time-dependent comparison of the amount of PTEN staining in the oocyte cytoplasm and granulosa cells of the follicles at different stages of development was made. While the follicles were developing from the primordial follicle to the primary and antral follicle, the amount of PTEN staining in the oocyte cytoplasm decreased, whereas the PTEN activity in the granulosa cells increased as the oocyte developed (p = 0.001 and p = 0.001, respectively).

**Table 4:** A comparative analysis of time-dependent PTEN staining levels in oocyte cytoplasm and granulosa cells across various stages of follicular development.

Variables	Time	PTEN staining levels					
		Mean ± SD	Median (Min–Max)	p values for time 1–2	p values for time 1–3	p values for time 2–3	Overall p value
PTEN immunoreactivity scores in oocyte cytoplasm	Time 1	3.00 ± 0.00	3.00 (3.00–3.00)	0.076	< 0.001	0.001	0.001
	Time 2	2.50 ± 0.55	2.50 (2.00–3.00)				
	Time 3	1.17 ± 0.41	1.00 (1.00–2.00)				
PTEN immunoreactivity scores in granulosa cells	Time 1	1.17 ± 0.41	1.00 (1.00–2.00)	0.076	< 0.001	0.001	0.001
	Time 2	1.67 ± 0.52	2.00 (1.00–2.00)				
	Time 3	3.00 ± 0.00	3.00 (3.00–3.00)				

Time 1, primordial follicle stage; Time 2, primary follicle stage; Time 3, antral follicle stage.

SD, standard deviation; Min, minimum; Max, maximum.

PTEN immunoreactivity scores were evaluated using an immunoreactive scoring scale, a total of six zones, consisting of one core zone and five outer zones, were chosen from the sections of ovarian tissue.

## Discussion

In this study, PCOS was modelled with letrozole in adult female Sprague‒Dawley rats, and PTEN expression in ovarian tissues was compared between the PCOS group and the normo-ovulatory (control) group. The results of our study indicate that there was a significant rise in PTEN staining levels in the oocytes of the PCOS group compared to the control group. This study additionally examined the alterations in PTEN expression within the ovary across various stages of folliculogenesis. The study revealed that PTEN expression became more prominent in granulosa cells as the follicles developed, whereas its presence in the oocyte cytoplasm decreased.

Hormonal and biochemical assessments, as well as follicle counting sections, were incorporated into the study to provide additional support for the development of the PCOS model. The utilisation of letrozole-induced rat models for the study of PCOS is widely favoured in the academic literature due to the notable matching of ovarian morphology and hormonal profiles to those observed in human PCOS^17–19^. The hormonal, biochemical, and histopathological analyses of the PCOS group in our study were comparable to those of human PCOS. In the PCOS group of the study, the observed reduction in primordial follicles, along with an elevation in primary and secondary follicles, may suggest atypical premature activation and commencement of development in primordial follicles. Alternatively, it is plausible that these findings could be attributed to potential discrepancies in the methodology employed for follicle quantification.

The histopathological analyses revealed a decrease in the number of granulosa cells within the follicles of individuals with polycystic ovary syndrome (PCOS) compared to that of the control group. Additionally, the arrangement of these cells appeared to be more loosely organised. The data presented in this study demonstrate a correlation between decreased ovulation and impaired granulosa cell function, despite an observed increase in the number of antral follicles. Furthermore, the ovaries of the PCOS group had many cystic forms and the absence of corpus luteum structures, which can be attributed to anovulation.

Based on the existing scientific evidence, the mechanism governing the proliferation of granulosa cells is highly intricate and is acknowledged to be intricately linked to numerous elements^23^. A growing body of literature has demonstrated that PTEN exerts regulatory control over the differentiation process in granulosa cells and is important in the aberrant proliferation of granulosa cells in polycystic ovaries^24,25^. In a study conducted by Yeung et al., the researchers examined the impact of PCOS on women by investigating PTEN expression in rat ovaries. The findings revealed a decrease in PTEN expression in PCOS-affected ovaries, leading to the hypothesis that PTEN may play a role in diminishing the proliferative potential of granulosa cells in polycystic ovaries. Consequently, this mechanism may contribute to the progression of follicular apoptosis and follicular atresia^26,27^. In the investigation conducted by Andreas et al.^28^, it was proposed that the decrease in PTEN expression leads to an augmentation in the proliferation of bovine granulosa cells. The variations observed in the outcomes of studies documented in the literature can be ascribed to the diverse origins of the granulosa cells utilised. Additionally, disparities may arise from the evaluation of distinct stages of cell development. Notably, the sources of these cells encompass rats, cattle, and female subjects, while the stages of follicle development examined encompass the primordial, primary, and antral stages. Hence, we conducted an investigation to analyse the fluctuation of PTEN expression during various developmental stages, with a focus on temporal dynamics. Our work revealed the presence of variation in PTEN expression inside both cytoplasmic and granulosa cells. Our study provides insights into the literature in this regard.

The primary constraint of our study is the limited sample size of rats employed in the research. The limited number of rats used in each study group was permitted by our ethics committee. A further constraint of this work pertains to the exclusive demonstration of time-dependent alterations in PTEN expression within follicular developmental stages solely in the control group. This particular aspect of the study was not conducted because the PCOS group did not have oestrous cycles and was deemed to be unable to ovulate.

There has been an emerging trend in recent years in the literature on PCOS and PTEN, wherein researchers have begun to challenge the plausibility of regulating PTEN expression. Researchers conducted a study to examine the fundamental mechanisms that contribute to the excessive proliferation observed in granulosa cells affected by PCOS. In this study, He et al., presented evidence indicating that miR-200b and miR-200c exert inhibitory effects on the proliferation of KGN cells through the targeting of PTEN. These findings contribute to the existing knowledge on the aberrant proliferation of granulosa cells in PCOS^15^. Zhou et al., investigated whether microRNA (miR)-18b-5p is one of the miRs that could functionally mediate PCOS progression via PTEN in rats that developed PCOS with letrozole, as in our study. miR-18b-5p targeted PTEN, reduced PTEN expression and activated the PI3K/Akt/mTOR signalling pathway to improve PCOS. As a result of this study, it was shown that circulating miR-18b-5p levels can contribute to the progression of PCOS complications^29^.

The objective of ongoing investigations on the PI3K/AKT/PTEN signalling system in polycystic ovary syndrome (PCOS) is to ascertain the viability of a pharmacological intervention capable of effectively addressing ovarian dysfunction^30^. Onal et al.^31^ conducted a recent study to determine whether PCOS treatment agents alter PTEN expression in the polycystic ovary, and the study highlighted the importance of PTEN pathway studies for PCOS treatment research groups.

Ongoing research is being conducted to investigate the importance of PTEN expression in polycystic ovary syndrome. Additionally, there is a parallel focus on examining the various elements that exert an influence on PTEN activity. The findings from these studies have paved the way for potential future therapies that can effectively modulate the mechanisms behind follicle and oocyte growth in patients with PCOS. The ability to modulate uncontrolled follicular development in patients with PCOS or the atretic process during follicular developmental stages holds great potential for minimising difficulties associated with PCOS and may have substantial implications, particularly in the field of assisted reproductive techniques.

Our research is anticipated to have a major impact on the existing body of literature, as it uncovers alterations in PTEN expression during the process of normal oocyte development, as well as diminished ovarian functions observed in those with PCOS.

## Conclusion

The present study investigated the alterations in PTEN ovarian tissue expression during the process of normal folliculogenesis as well as in cases of impaired folliculogenesis, with a specific focus on rats with PCOS. The results of our study will provide a basis for further research on the relationship between PTEN and the processes of folliculogenesis, both in normal and disturbed contexts, particularly in PCOS.

## Data Availability

The datasets utilised in this investigation may be obtained from the corresponding author upon a reasonable request.

## References

[1] Skiba MA, Islam RM, Bell RJ, Davis SR (2018). Understanding variation in prevalence estimates of polycystic ovary syndrome: A systematic review and meta-analysis. Hum. Reprod. Update.

[2] Yildiz BO, Bozdag G, Yapici Z, Esinler I, Yarali H (2012). Prevalence, phenotype and cardiometabolic risk of polycystic ovary syndrome under different diagnostic criteria. Hum. Reprod..

[3] Rotterdam ESHRE/ASRM-Sponsored PCOS Consensus Workshop Group (2004). Revised 2003 consensus on diagnostic criteria and long-term health risks related to polycystic ovary syndrome (PCOS). Hum. Reprod..

[4] Azziz R (2016). New insights into the genetics of polycystic ovary syndrome. Nat. Rev. Endocrinol..

[5] Webber L, Stubbs S, Stark J, Trew G, Margara R, Hardy K, Franks S (2003). Formation and early development of follicles in the polycystic ovary. The Lancet.

[6] Matsuda F, Inoue N, Manabe N, Ohkura S (2012). Follicular growth and atresia in mammalian ovaries: Regulation by survival and death of granulosa cells. J. Reprod. Dev..

[7] Munakata Y, Kawahara-Miki R, Shiratsuki S, Tasaki H, Itami N, Shirasuna K, Kuwayama T, Iwata H (2016). Gene expression patterns in granulosa cells and oocytes at various stages of follicle development as well as in in vitro grown oocyte-and-granulosa cell complexes. J. Reprod. Dev..

[8] Gu L, Liu H, Gu X, Boots C, Moley KH, Wang Q (2015). Metabolic control of oocyte development: Linking maternal nutrition and reproductive outcomes. Cell. Mol. Life Sci..

[9] Dewailly D, Robin G, Peigne M, Decanter C, Pigny P, Catteau-Jonard S (2016). Interactions between androgens, FSH, anti-Müllerian hormone and estradiol during folliculogenesis in the human normal and polycystic ovary. Hum. Reprod. Update.

[10] Yin Y, Shen WH (2008). PTEN: A new guardian of the genome. Oncogene.

[11] Feng X-J, Liu S-X, Wu C, Kang P-P, Liu Q-J, Hao J, Li H-B, Li F, Zhang Y-J, Fu X-H (2014). The PTEN/PI3K/Akt signaling pathway mediates HMGB1-induced cell proliferation by regulating the NF-κB/cyclin D1 pathway in mouse mesangial cells. Am. J. Physiol.-Cell Physiol..

[12] Li Q, He H, Zhang Y-L, Li X-M, Guo X, Huo R, Bi Y, Li J, Fan H-Y, Sha J (2013). Phosphoinositide 3-kinase p110δ mediates estrogen-and FSH-stimulated ovarian follicle growth. Mol. Endocrinol..

[13] Tu J, Cheung H-H, Lu G, Chen Z, Chan W-Y (2018). MicroRNA-10a promotes granulosa cells tumor development via PTEN-AKT/Wnt regulatory axis. Cell Death Dis..

[14] Courtney KD, Corcoran RB, Engelman JA (2010). The PI3K pathway as drug target in human cancer. J. Clin. Oncol..

[15] He T, Sun Y, Zhang Y, Zhao S, Zheng Y, Hao G, Shi Y (2019). MicroRNA-200b and microRNA-200c are up-regulated in PCOS granulosa cell and inhibit KGN cell proliferation via targeting PTEN. Reprod. Biol. Endocrinol..

[16] Hsueh AJ, Kawamura K, Cheng Y, Fauser BC (2015). Intraovarian control of early folliculogenesis. Endocrine Rev..

[17] Li C, Chen L, Zhao Y, Chen S, Fu L, Jiang Y, Gao S, Liu Z, Wang F, Zhu X (2017). Altered expression of miRNAs in the uterus from a letrozole-induced rat PCOS model. Gene.

[18] Baravalle C, Salvetti NR, Mira GA, Pezzone N, Ortega HH (2006). Microscopic characterization of follicular structures in letrozole-induced polycystic ovarian syndrome in the rat. Arch. Med. Res..

[19] Kafali H, Iriadam M, Ozardalı I, Demir N (2004). Letrozole-induced polycystic ovaries in the rat: A new model for cystic ovarian disease. Arch. Med. Res..

[20] Feldman AT, Wolfe D (2014). Tissue processing and hematoxylin and eosin staining. Histopathol. Methods Protoc..

[21] Gundersen G, Hans-Jørgen G, Jensen EB (1987). The efficiency of systematic sampling in stereology and its prediction. J. Microsc..

[22] Gougeon A (1996). Regulation of ovarian follicular development in primates: Facts and hypotheses. Endocrine Rev..

[23] Kranc W, Budna J, Kahan R, Chachuła A, Bryja A, Ciesiółka S, Borys S, Antosik M, Bukowska D, Brussow K (2017). Molecular basis of growth, proliferation, and differentiation of mammalian follicular granulosa cells. J. Biol. Regul. Homeost. Agents.

[24] Chen M-J, Chou C-H, Chen S-U, Yang W-S, Yang Y-S, Ho H-N (2015). The effect of androgens on ovarian follicle maturation: Dihydrotestosterone suppress FSH-stimulated granulosa cell proliferation by upregulating PPARγ-dependent PTEN expression. Sci. Rep..

[25] Iwase A, Goto M, Harata T, Takigawa S, Nakahara T, Suzuki K, Manabe S, Kikkawa F (2009). Insulin attenuates the insulin-like growth factor-I (IGF-I)-Akt pathway, not IGF-I-extracellularly regulated kinase pathway, in luteinized granulosa cells with an increase in PTEN. J. Clin. Endocrinol. Metabol..

[26] Yeung CK, Wang G, Yao Y, Liang J, Tenny Chung CY, Chuai M, Lee KKH, Yang X (2017). BRE modulates granulosa cell death to affect ovarian follicle development and atresia in the mouse. Cell Death Dis..

[27] He T, Liu Y, Zhao S, Liu H, Wang Z, Shi Y (2019). Comprehensive assessment the expression of core elements related to IGFIR/PI3K pathway in granulosa cells of women with polycystic ovary syndrome. Eur. J. Obstetr. Gynecol. Reprod. Biol..

[28] Andreas E, Hoelker M, Neuhoff C, Tholen E, Schellander K, Tesfaye D, Salilew-Wondim D (2016). MicroRNA 17–92 cluster regulates proliferation and differentiation of bovine granulosa cells by targeting PTEN and BMPR2 genes. Cell Tissue Res..

[29] Zhou Z, Tu Z, Zhang J, Tan C, Shen X, Wan B, Li Y, Wang A, Zhao L, Hu J (2022). Follicular fluid-derived exosomal MicroRNA-18b-5p Regulates PTEN-mediated PI3K/Akt/mTOR signaling pathway to inhibit polycystic ovary syndrome development. Mol. Neurobiol..

[30] Xie F, Zhang J, Zhai M, Liu Y, Hu H, Yu Z, Zhang J, Lin S, Liang D, Cao Y (2021). Melatonin ameliorates ovarian dysfunction by regulating autophagy in PCOS via the PI3K-Akt pathway. Reproduction.

[31] Onal T, Tulay P, Vatansever H (2023). Does Pten have an impact on oogenesis of PCOS mouse models?. Zygote.

